# Dibutyl sebacate as PVC ecoplasticizer—economic synthesis and functional properties

**DOI:** 10.1098/rsos.241984

**Published:** 2025-03-26

**Authors:** Anna Tracz, Sławomir Boncel, Ewa Pankalla, Anna Chrobok

**Affiliations:** ^1^ Faculty of Chemistry, Department of Organic Chemistry, Bioorganic Chemistry and Biotechnology, NanoCarbon Group, Silesian University of Technology, Gliwice 44-100, Poland; ^2^ Grupa Azoty Zakłady Azotowe Kędzierzyn S.A., Kędzierzyn-Koźle 47-220, Poland; ^3^ Centre for Organic and Nanohybrid Electronics (CONE), Silesian University of Technology, Gliwice 44-100, Poland; ^4^ NanoCarbonGroup.com Ltd., Gliwice 44-100, Poland; ^5^ Faculty of Chemistry, Department of Organic Chemistry, Bioorganic Chemistry and Biotechnology, NanoCarbon Group, Silesian University of Technology, Gliwice 44-100, Poland; ^6^ Faculty of Chemistry, Department of Chemical Organic Technology and Petrochemistry, Silesian University of Technology, Gliwice 44-100, Poland

**Keywords:** alkyl esters, plasticizers, poly(vinyl chloride), Fischer synthesis, mechanical properties

## Abstract

One-third of poly(vinyl chloride) (PVC) applications require plasticization to improve flexibility, softness, and processability. Phthalate esters have been widely used but are now restricted due to their toxicity. Di-*n*-butyl sebacate (DBS), a safe, biodegradable, and cost-effective aliphatic ester, offers superior operational properties and industrial scalability compared to phthalates. We demonstrate a scalable DBS synthesis achieving approximately 100% yield under optimized conditions (90°C, 15 mol% triethylamine-sulfuric(VI) acid catalyst, 4 : 1 BuOH to sebacic acid ratio, 2 h). Kilogram-scale DBS-plasticized PVC was produced and evaluated for key properties. The DBS-plasticized PVC showed enhanced performance, including minimal plasticizer migration (12.78% after 28 days, per EN ISO 177:2017), high extension (350%), breaking stress of 15.7 MPa, and a Shore A hardness of 80.2. These results outperform conventional phthalates, such as di-2-ethylhexyl terephthalate and di-2-ethylhexyl phthalate. The findings confirm that DBS synthesis is fully scalable and its use results in PVC materials with superior mechanical and leakage properties. This study supports the industrial adoption of DBS as an eco-friendly and effective alternative to replace toxic phthalates in PVC plasticization, promoting safer and more sustainable materials for widespread applications.

## 1. Introduction

Poly(vinyl chloride) (PVC) products range from rigid to very flexible and from nondurable to long-lasting [1]. PVC is used in construction products such as pipes/fittings, window frames, sidings, flexible roofing membranes, and wire coatings in cables. It is also used in home and office floorings and as upholstery, wall coverings, and laminates of various types. On the other hand, PVC is widely used in short-lived products, including toys, fishing lures, wearing apparel, advertising banners, and signs [1]. While neat PVC is a rigid polymer, many industries require its flexible and processable form. Hence, the addition of a plasticizer makes the target plastics and composites softer and more flexible [2]. Indeed, one-third of the total produced PVC in the world is consumed as *plasticized* PVC [3]. Most often, plasticizers cause an increase in the flexibility and workability by a decrease in the glass transition temperature (*T*_g_) of the polymer matrix [4]. The plasticizer molecules diffuse between the polymer chains and keep them further apart, which reduces the attractive forces, making the material more flexible. Eventually, the polymer displays a reduced tensile strength and stiffness as well as enhanced processability. PVC products require a bulk quantity of plasticizers that could alter their thermal and mechanical properties [5]. Di(2-ethylhexyl)phthalate (‘dioctyl phthalate’) (DEHP or DOP) and di-*n*-butyl phthalate (DBP) are commonly used as commercial plasticizers for PVC products [3].

**Figure 1.**
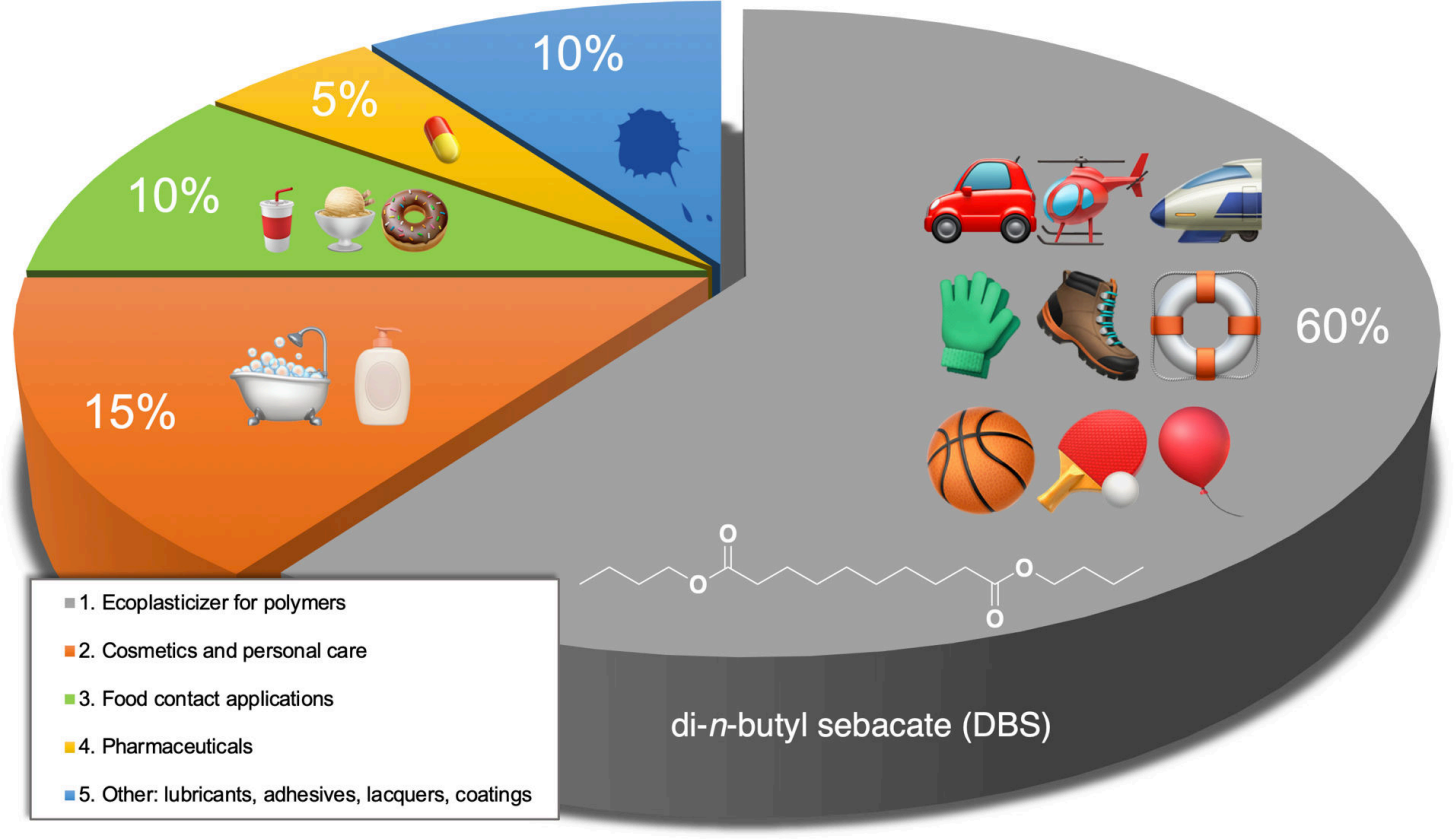
Large-scale applications of DBS, prepared on the basis of data in [13].

Typically, plasticizers are non-covalently bound to the polymers that leads to their leakage over time, resulting in human exposure and environmental contamination. Phthalates, in particular, have been the subject of increasing concern due to their ubiquity in the environment and negative health effects, including endocrine-disrupting and anti-androgenic effects [6]. Therefore, phthalates have gradually been restricted because of their migration and reproductive toxicity [7]. Nevertheless, DOP remains an international standard of PVC plasticizers, i.e., specifications to other plasticizers are referenced to the specification of DOP [8]. In turn, *ecoplasticizers* could be useful for applications that are especially sensitive to migration and subsequent toxicity, such as the cable manufacturing industry, food packaging and children’s toys [9]. Sebacic acid (decanedioic acid) (SA), the key precursor of di-*n*-butyl sebacate (DBS), is primarily derived from castor oil with two primary uses: (i) as an important synthetic precursor of large-scale chemical compounds and polymers; and (ii) directly in formulated products such as corrosion inhibitors in cutting and metal-working fluids. SA can be used directly in formulated products such as antifreeze coolants [10]. Generally, sebacate esters are frequently used as liquid proxies for SA in making greases and compete with the chemical derivatives of adipic acid, azelaic acid and dodecanedioic acid as plasticizers [11]. Apart from PVC, sebacates are recommended plasticizers for poly(vinylidene dichloride), acrylics, ethyl cellulose, poly(methyl methacrylate), poly(vinyl acetate), polyvinyl butyral (PVB) as well as nitrile, neoprene, and chlorinated rubbers. The main applications of sebacates are frost-resistant cables, aircraft and car interior parts, flooring, films, paints, food wrap, pharmaceutical coatings, packaging materials, medical and synthetic leather with an excellent low-temperature flexibility [12]. Also, SA esters indicate excellent lubrication properties with high flash point and high viscosity index [11]. As for DBS specifically, its large-scale applications are summarized in figure 1.

DBS is mostly applied as (i) an ecoplasticizer of cellulose acetate butyrate/propionate, EC, PVB, PVC, polystyrene and synthetic rubbers—in food packaging, medical devices, drug coatings (in tablets, beads, and granules) and solvent in skincare formulations; (ii) lubricant in shaving lotions; and (iii) flavouring additive in non-alcoholic beverages, ice cream, candies, and baked goods.

The most effective plasticizers achieve an increase in the free volume accessible for the polymer chains by a relatively large branched molecular structure but at low molecular weight. Specifically, the ester groups in plasticizers act as cohesive moieties due to van der Waals forces, hydrogen bonding, and electrostatic interactions that dominate their interactions with the PVC backbone [14]. Indeed, thermomechanical properties of non-toxic PVC plasticizers predicted by molecular dynamics simulations indicated that DBS had properties comparable to DEHP except its toxicity [14]. DBS was also indicated as one of the most effective plasticizers among the family of sebacates [15]. It has decent low-temperature properties, has low volatility, and is physiochemically compatible with PVC. Generally, to estimate the physicochemical compatibility of DBS and PVC, the Hansen solubility parameters (HSP) should be compared. And so, dispersion, polar, and hydrogen bond forces components for PVC versus DBS are equal to *δ*_d_ = 16.8 MPa^0.5^, *δ*_p_ = 8.9 MPa^0.5^ and *δ*_h_ = 6.1 MPa^0.5^ versus *δ*_d_ = 16.7 MPa^0.5^, *δ*_p_ = 4.5 MPa^0.5^ and *δ*_h_ = 4.1 MPa^0.5^, respectively [16]. Hence, since for none of the HSP values are the differences higher than 5 MPa^0.5^, the comparison suggests the excellent solubility of DBS in the PVC matrix, which was confirmed experimentally. Importantly, to the best of our knowledge, the tensile properties of PVC plasticized with sebacates have not been previously studied, while Mahnaj *et al*. investigated their effectiveness in ethylcellulose polymer [17]. DBS as a promising plasticizer was described already in 1945 [18], but it was somewhat forgotten over the years. Moreover, the data on processing studies for the DBS and PVC system are rather scarce [12].

Recently, several publications on the synthesis and use of alternative PVC plasticizers have appeared. For instance, diester plasticizers based on suberic, azelaic, and sebacic acids were synthesized by reacting them with 2-ethylhexanol by Fischer esterification [19]. Also, epoxidized and acylated (acyl and benzoyl) castor oil by 30% H_2_O_2(aq)_ in the presence of cation exchange resin catalyst and acetyl anhydride or benzoyl chloride under basic conditions, respectively [20], and epoxidized (30% H_2_O_2(aq)_, HCOOH) mixed esters of succinic and oleic acid and propylene glycol (via Fischer reaction in the presence of methanesulfonic acid) efficiently plasticize PVC [21]. All of the above mentioned led to plasticized PVC films of enhanced mechanical performance in comparison with the traditional commercially available phthalate plasticizers. Other PVC bioplasticizers, with operational characteristics similar to the conventional phthalates, methyl or ethyl 10-(2-methoxy-2-oxoethanesulfonyl) octadecanoate were synthesized from oleic acid via, in sequence, UV-assisted addition of thioglycolic acid to the C=C bond, oxidation of the thioether group using 30% H_2_O_2(aq)_ in ethyl acetate, and Fischer esterification with MeOH or EtOH [22]. Additionally, a new green plasticizer based on a terminally acetylated ester of oligomeric L-lactic acid and 1,6-hexanediol – again formed via Fischer esterification in the presence of *p*-toluenesulfonic acid – was also synthesized. This composition of a molecular weight in the range from 200 to 800 Da has proved, as compared to the commercial plasticizer acetyltributylcitrate (ATBC), its better resistance to organic solvents and higher stability in the food-mimicking migration studies on PVC foils [23].

Synthesis of SA esters is mainly performed via Fischer esterification in the presence of strong Brønsted acid catalysts such as sulfuric(VI) or *p*-toluenesulfonic acid. However, such an approach involving homogeneous catalysis is not environmentally benign and requires costly neutralization upon inefficient catalyst separation from the post-reaction mixtures. This characteristic results in a substantial consumption of energy and production of large amounts of dangerous waste [24]. In the synthesis of long-chain aliphatic esters for cosmetics, solid–liquid solvent-free phase transfer catalysis, and acidic catalysis can be applied under both conventional and microwave heating, achieving high yields in short times [25]. *n*-Butyl acetate [26] and a range of alkyl levulinates [27] were synthesized under mild conditions (room temperature, atmospheric pressure) using inexpensive and efficient Brønsted acidic ionic liquids (ILs) based on sulfuric(VI) acid and off-the-shelf bases. Inexpensive Brønsted acidic ILs based on trimethylamine and sulfuric acid were also proposed as both solvents and catalysts in the synthesis of bis(2-ethylhexyl) terephthalate [28,29].

Here, we present a heterogeneous catalytic approach towards the synthesis of DBS as a promising PVC ecoplasticizer. The optimized conditions allowed for a quantitatively scalable synthesis of DBS with extraction of the two-phase post-reaction mixture as economic and convenient. The operational characteristics of the product were compared against the ‘golden standards’ but ‘substitute-it-now’ phthalates showed the superior figure-of-merit of DBS embedded into PVC matrix. Despite advancements in the field of novel PVC plasticizers as alternatives to phthalates, the existing synthetic routes are not always green or economically viable. This paper introduces a 100% yield synthesis of DBS under benign conditions, further demonstrating its superior functionality to well-established but dangerous dioctyl terephthalate (DEHT) and DEHP.

## 2. Experimental

### 2.1. Materials

Triethylamine (>99%), sulfuric(VI) acid (95%), and *n*-decane (>99%) were purchased from Sigma-Aldrich (Darmstadt, Germany). SA (98%) was purchased from Acros Organics (Geel, Belgium). *n*-Butanol was produced at the plants of Grupa Azoty Zakłady Azotowe Kędzierzyn S.A. (Kędzierzyn-Koźle, Poland). Suspension PVC Polanvil PVC-S70 was purchased from Anvil S.A. (Włocławek, Poland). A PVC stabilizing additive Baeropan R 8890 KA/2 (Ca/Zn) was purchased from Baerlocher GmbH (Unterschleissheim, Germany), while Chalk Extra 1 (calcium carbonate) from Piotrowice Ltd. (Piotrowice, Poland).

### 2.2. Synthesis of the ionic liquid catalyst

Synthesis of the IL catalyst is described in detail in the electronic supplementary material.

### 2.3. Synthesis of DBS

**Figure 2.**
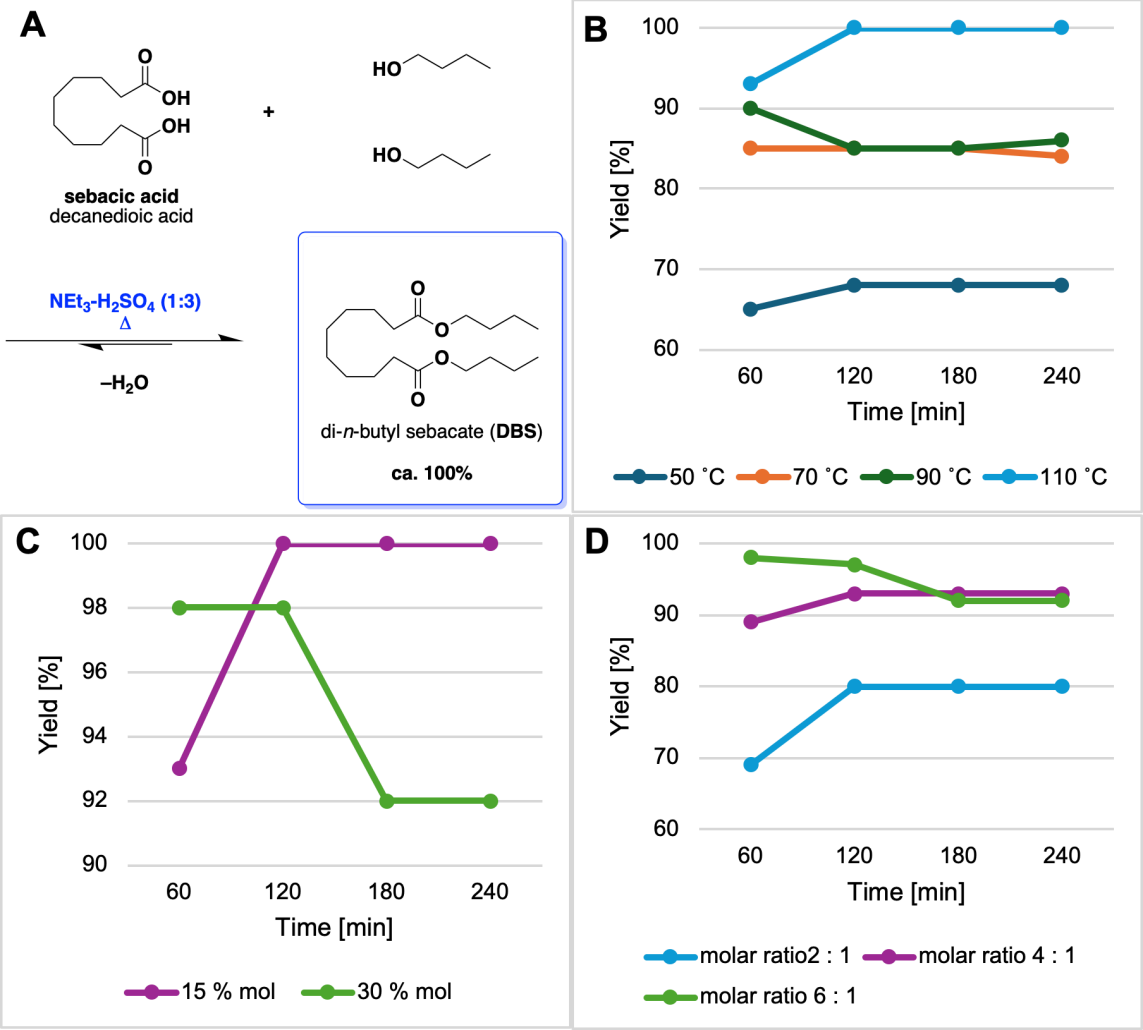
(A) Scheme of the reaction of synthesis of DBS. Progress of the Fischer esterification of SA with *n*-BuOH: (B) the effect of temperature; *n*-BuOH : SA = 6 : 1 mol/mol, *m_n_*_-BuOH_ = 4.3907 g, *m*_SA_ = 2.0000 g, 15% mol cat (3.0) IL/mol SA, 1000 rpm; (C) the effect of the catalyst amount; *n*-BuOH : SA = 6 : 1 mol/mol, *m_n_*_-BuOH_ = 4.3907 g, *m*_SA_ = 2.0000 g, *T* = 110°C, 1000 rpm, (D) the effect of the molar ratio of reagents; *m*_SA_ = 2.0000 g, 30% mol cat (3.0) IL/mol, *T* = 110°C, 1000 rpm. Each experiment was performed in duplicate. Each measurement point was generated from four GC measurements revealing the relative error <5% as a standard deviation. The eye-guiding lines on the chart are only for the purpose of illustrating trends/patterns.

DBS was synthesized under various conditions, further presented in figure 2. Isolation of DBS was performed using an extraction of a two-phase post-reaction system. The mixture was composed of the top layer containing DBS and the excess alcohol with a small amount of ionic liquid, while the bottom phase contained solely ionic liquid and water. The upper layer was extracted several times with small volumes of *n*-hexane (in total 20 ml) and water (3 × 20 ml) to pH = 7. The extract was dried using anhydrous MgSO_4_, and the filtrate was distilled to obtain the ester. *n*-BuOH was removed by distillation at 118°C (1 atm). DBS was obtained as a colourless liquid of 96.5% purity as determined by GC. Eventually, the scale of the di-*n*-butyl sebacate synthesis was increased 20-fold relative to the amount of carboxylic acid used, yielding the product of practically identical properties/purity.

### 2.4. GC analysis

GC analyses were performed on an Agilent Technologies 8890 (Agilent Technologies, Santa Clara, United States) with He as a carrier gas. The chromatograph was equipped with a flame ionization detector and an HP-5 column 19091 J-413 (30 m × 320 µm × 0.25 µm). The GC oven was set to the following parameters: 70°C for 3 min, then a ramp 30°C min^–1^ and 240°C for 7 min. Temperature of an injector and the detector was 220 and 280°C, respectively. Split injection parameters were as follows: aplit ratio 100 : 1, split flow 127.34 ml min^–1^, syringe size 10 µl, injection volume 1 µl. The carrier gas was He (221.43 ml min^–1^), while combustion gases were H_2_ (30 ml min^–1^) and air (400 ml min^–1^). At the above conditions, retention times (*R*_f_) of *n*-BuOH, *n*-decane, and DBS were found to be 1.921, 4.801, and 10.568 min, respectively.

### 2.5. Manufacturing of a dry-blend mixture

In order to compare plasticizers, the same amounts of plasticizer, stabilizer, chalk, and PVC were used. In each experiment, the limiting amounts of plasticizer were used, up to 50 parts per hundred resin. A formula for the manufacturing of plasticized PVC plastics was as follows: PVC (1094 g, 100 weight parts (wp)), plasticizer (547 g, 50 wp), stabilizer (49 g, 4.5 wp), chalk (109 g, 10 wp). The components were mixed using a set of Labtech Engineering mixers type LMX5I, LCM-12 to obtain 1.800 kg of a dry-blend mixture. As first verified, pre-adsorption of DBS by chalk emerged as insignificant.

The dry-blend mixture production process had two basic parameters: the blade rotation speed and the temperature of the mixture generated by rubbing the blade against the backfilled raw material. An additional parameter, equally important, was the resistance provided by the mixture to the rotating blade, i.e., the torque needed to overcome the mixture resistance at a constant blade rotation speed. The parameters for obtaining the herein-studied mixtures were developed experimentally and were subjected to further processing in order to assess the utility of the plasticizer. And the final, actual processing was modelled on it. The following processes were observed during mixing. For the temperature range up to 80°C, PVC mixtures with thermal stabilizer were heated to the required temperature, at which the plasticizer was dosed, using a rotating stirrer in the mixer. In the temperature range from 80°C to 95°C, the plasticizer was added at 80°C. With the increase in temperature, the viscosity of the mixture also increased. This was the result of the absorption of the plasticizer by the PVC grain, which increased its volume at the same time as creating larger agglomerates with other grains in the initial phase. With a further increase in temperature, an increase in resistance was observed on the stirrer blades. This indicated the ongoing process of grain plasticization. In the final phase of this stage, a slight decrease in torque was recorded, which meant that it was necessary to proceed to the next stage—adding the chalk filler. In the temperature range from 95°C to 110°C, chalk was added at 95°C. This resulted in the ‘drying’ of the mixture, where larger agglomerations of glued grains disintegrated to form the appropriate mixture. As a result of adding chalk, there was also an increase in mixing resistance in this temperature range. The disintegration of larger agglomerations of grains into the final form of the dry-blend mixture (much smaller grain clusters, many individual grains) also resulted in an increase in the mixing resistance. In the temperature range from 105°C to 110 °C, there was a slight decrease in resistance on the mixer. The process of plasticization of PVC grains was completed, and the dry-blend mixture obtained its target form. The mixture was left for 24 h with the bag/container open. Then, the product was extruded and granulated. Extrusion and granulation of a dry blend were performed using a Zamak Mercator granulation line with an RES-2P/24 A twin-screw extruder.

### 2.6. Migration studies

Compression of auxiliary low-density polyethylene (LDPE) sheets and PVC sheets was carried out by a press (Labtech Engineering Company Ltd., Samutprakarn, Thailand) with a maximum pressure of 160 bar and a compression force of 30 tonnes. Sample conditioning prior to the physicochemical testing was performed using a climate chamber, type KBF P240 (BINDER GmbH, Tuttlingen, Germany), maintaining uniform climatic conditions. The samples were conditioned in the chamber at 23°C and a humidity of 50% RH. The samples dedicated to the migration test were cut using a pneumatic press (ZwickRoell, Wroclaw, Poland) and the appropriate cutting die. The migration test was performed on the basis of the standard, i.e., *Plastics—Determination of the migration of plasticizers* (EN ISO 177:2017). Briefly, the standard describes a method for determining the tendency of plasticizers to migrate from plastics in which they are diffusively transferred to other materials or to other plastics in contact. The test sample is placed in close contact with two sheets capable of absorbing softeners. It is then heated under specific conditions. The loss of mass of the test sample is theoretically equal to the increase in mass of the absorbent sheets as a measure of the plasticizer migration. Here, a sample of the tested material (plasticized PVC) was placed between two absorbent LDPE discs so that their axes overlapped, and the set formed a sandwich arrangement. It was placed between two glass plates. Such a system (the set of discs and test plates) was weighted with 5 kg weights and was placed in an oven at 70 ± 2°C. To determine the migration progress as a function of time, the measurements were taken after the following times: 2, 4, 7, and 28 days. The migration test was performed in a 1/2/1 system, i.e., the stack consisted of 1 mm LDPE disc with a diameter of 60 mm, a 2 mm PVC disc with a diameter of 50 mm and a 1 mm LDPE disc with a diameter of 60 mm. Shore A hardness tests were performed on a hardness tester (ZwickRoell, Wroclaw, Poland). The hardness of polymeric material was recorded by the Shore method in accordance with the PN-ISO 868 standard, with an indenter in accordance with the PN-93/C-04206 standard (type A hardness testers are used for soft materials, and types C and D for hard materials). Tensile strength tests were carried out using a static testing machine type Z010TE (ZwickRoell, Wroclaw, Poland). The quantities measured in this test are deformation (elongation) and deformation force. The testing speed was 100 mm min^–1^. The distance of the grips at the starting position was 57.21 mm.

## 3. Results and discussion

In order to optimize the synthesis of DBS via Fischer esterification (figure 2), i.e., from SA (constant amount 2.00 g) and *n*-BuOH with *n*-decane as the solvent and the GC marker (0.20 g) (figure 2A), a series of experiments was performed under conditions with varying parameters: temperature and duration time (figure 2B), the amounts of catalyst relative to SA (15 and 30 mol%) (figure 2C), and the molar excess (twofold, fourfold, and sixfold) of *n*-BuOH in relation to SA (figure 2D).

Apart from DBS as the main reaction product, mono-*n*-butyl sebacate and water were identified as the intermediate and side product, respectively. To shift the equilibrium toward formation of the product, the excess of *n*-BuOH (as the less expensive substrate) was used, while water was taken up by the IL catalyst. This behaviour led to a formation of the two-phase system: IL catalyst and water were found to be immiscible with DBS enabling its straightforward separation. The reactions were carried out at 50, 70, 90, and 110°C, revealing that a 100% yield of DBS can be obtained by conducting the reaction for 120 min at 110°C (after 60 min and at 90°C, a still high yield of >90% was observed), while lower temperatures had an unfavourable effect on the course of the reaction. The NEt_3_-H_2_SO_4_ IL system, in addition to its catalytic function, also acted as a water absorbent, so it was important to determine the amount that should be used to ensure the highest possible efficiency. The studies, involving 15 and 30 mol% of the catalyst in relation to the amount of SA, showed that both amounts allowed a yield of DBS as high as 98−100% to be achieved. The influence of the molar ratio of substrates on the course of the model reaction was also verified using molar ratios of *n*-BuOH : SA = 2 : 1, 4 : 1 and 6 : 1, indicating a sixfold excess of *n*-BuOH as the most prospective one.

### 3.1. DBS as the PVC ecoplasticizer

**Figure 3.**
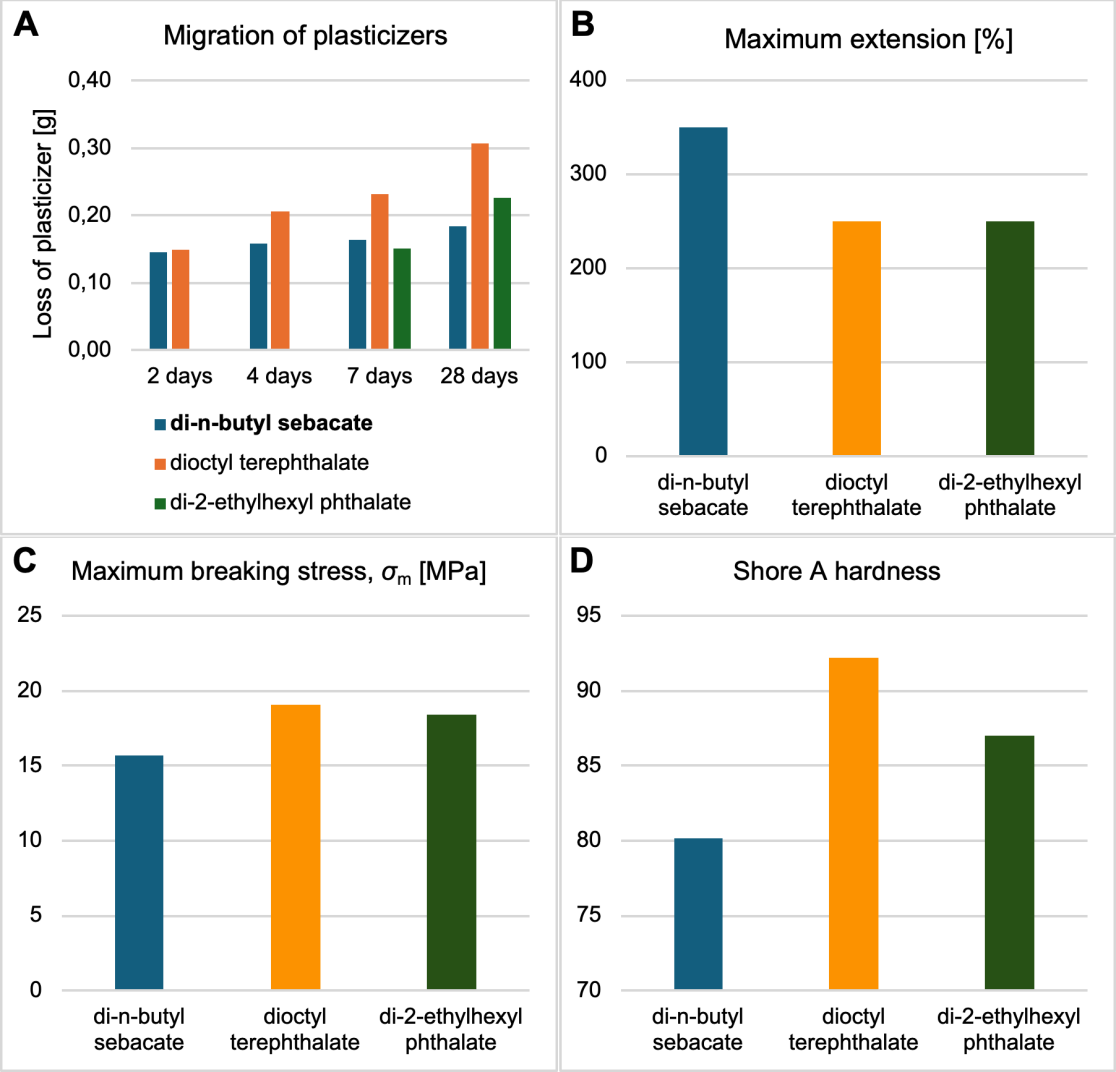
Comparison of operational parameters of the target plastics containing DBS with dioctyl terephthalate and di-2-ethylhexyl phthalate as the reference compounds: migration from the target plastics after 2, 4, 7, and 28 days (A), maximum elongation (%) (B), tensile strength (*σ*_m_) (MPa) (C), and Shore A hardness (D). The samples were studied in triplicate for the given property, with the differences not higher than 5% relatively calculated as standard deviations.

In the next step, we have cross-verified the functional characteristics of DBS as a PVC ecoplasticizer, in the background of standard phthalates, i.e., di-2-ethylhexyl terephthalate = DEHT and DEHP (figure 3).

Figure 3A presents the results of the plasticizer migration tests after 2, 4, 7, and 28 days. The largest weight loss after 28 days of the test was observed for DEHT, i.e., 0.31 g, while the smallest one for DBS 0.18 g, which corresponds to 12.78% of the initial amount of plasticizer in the sample. Our study generally corresponds to Castle *et al*. who carried out a survey of plasticizer levels in retail foods wrapped in plasticized films or materials with plasticized coatings [30]. Migration of DBS in processed cheese and cooked meats was found to be 76−137 mg kg^–1^, which was much lower than for DBP, dicyclohexyl phthalate, butylbenzyl phthalate, and diphenyl 2-ethylhexyl phosphate [30]. Hence, as for the growing demand for low-migration and non-toxic plasticizers alternative to phthalates, DBS emerges as one the most prospective compound [15].

Figure 3B shows the studies on the average elongation (%) upon tensile, which shows the highest value for DBS equal to 350% with the remaining plasticizers displaying elongation of *ca* 250%. At the same time, the maximum stress at break was recorded as the lowest one for DBS (15.7 MPa), while for DEHT and DEHP, the parameters were 19.1 and 18.4 MPa, respectively (figure 3C). As a degree of plasticity of polymers is largely dependent on the physicochemical affinity driven by chemical composition, molecular weight, and functional groups of the plasticizer [31], the results obtained for DBS—having the longest and the least polar aliphatic chains as compared to the other plasticizers—suggest it will generate more free volume and increase the plasticization [14].

Furthermore, the Shore A hardness for plasticized PVC samples was determined (figure 3D). For the DBS-plasticized PVC before the plasticizer migration test, the A hardness was found to be 80.2. After 2 days of the plasticizer migration test, due to the loss of a small amount of plasticizer (and water), it was changed to 82.5. Compared to the PVC samples plasticized with the reference plasticizers (92.2 and 87.0 Shore A for DEHT and DEHP, respectively), it is clear that the PVC samples with DBS were softer and more flexible. Importantly, the results corroborate tensile strength and elongation at break studies. Those correlations stem from the fact that softer PVC materials with lower hardness should display reduced tensile strength as the polymer matrix is less rigid and cohesive and higher extensibility as softer materials deform more before fracturing.

## 4. Conclusions and outlook

A protocol of scalable, highly efficient Fischer synthesis (approx. 100% yield) of DBS in the presence of inexpensive acidic IL was elaborated and optimized, with its readily extensibility toward other esters. The low-cost method covers a simple extraction-based DBS separation from the post-reaction mixture. *En route* to the large-scale applicability, polymer blends with DBS as the ecoplasticizer indicate its usefulness with the characteristics superior to the standard phthalates. Overall, DBS emerges as a compound, which synthetic pathway and physicochemistry fulfil the green chemistry criteria. DBS itself can be considered ecosolvent, ecolubricant, and ecoadditive to food, medicines and fragrances.

Over the past decade, PVC plasticized with DEHP has been one of the largest-scale plastics used in numerous commercial applications, such as cables, toys, baby products, medical products, packaging materials, and others [32]. Currently, an extensive research is underway to find plasticizers alternative to toxic phthalates. Nevertheless, DEHP remains an international standard of PVC plasticizers, i.e., specifications to other plasticizers are pre-set with its specification [8]. In this work, we have compared operational performance of DBS to the commercial phthalates: DEHP and DEHT. Our results indicated DBS as the PVC plasticizer with the superior properties. Obviously, it should be emphasized that changing the type and amount of plasticizer will affect the properties of the final plastic product [31]. The key aspect is that, generally, it is frequently difficult or even impossible to compare the existing data directly. However, for instance, molecular dynamics-based predictions indicated that DBS had properties comparable to or significantly better than DEHP, and our studies confirm this thesis [14]. In summary, our research suggests that the new synthetic approach to DBS with its characteristic may lead to a successful replacement of DEHP.

## Data Availability

The data supporting this article have been included as a part of the electronic supplementary material [33]. Any other data for the article cannot be made available due to the legal requirements, i.e., in connection with the current tripartite agreement between Grupa Azoty S.A. (Employer), Silesian University of Technology in Gliwice and Mrs. Anna Tracz guaranteeing the confidentiality of the obtained experimental data in order to implement the results of the doctoral thesis in the industrial production of the Employer. Supplementary material is available online [34].
